# The Triglyceride–Glucose Index Might Be a Better Indicator for Predicting Poor Cardiovascular Outcomes in Chronic Coronary Syndrome

**DOI:** 10.3390/jcm12196201

**Published:** 2023-09-26

**Authors:** Aslan Erdoğan, Duygu İnan, Ömer Genç, Ufuk Yıldız, Ayşe İrem Demirtola, İlyas Çetin, Yeliz Güler, Ali Fuat Tekin, Süleyman Barutçu, Ahmet Güler, Ali Karagöz

**Affiliations:** 1 Department of Cardiology, Basaksehir Cam & Sakura City Hospital, Istanbul 34480, Turkey; dr.duyguinan@gmail.com (D.İ.); dr.genc@hotmail.com (Ö.G.); drufukyildiz@gmail.com (U.Y.); airem90@gmail.com (A.İ.D.); drilyas@hotmail.com (İ.Ç.); yelizguler829@gmail.com (Y.G.); slybrtcu@gmail.com (S.B.); ahmetguler01@yahoo.com.tr (A.G.); 2 Department of Radiology, Basaksehir Cam & Sakura City Hospital, Istanbul 34480, Turkey; aftrad333@gmail.com; 3 Department of Cardiology, Kartal Kosuyolu Education and Training Hospital, Istanbul 34480, Turkey

**Keywords:** atherosclerosis, atherogenic index of plasma, chronic coronary syndrome, inflammation, triglyceride–glucose index

## Abstract

This study aimed to explore the potential association between the triglyceride–glucose index (TyG) and the atherogenic index of plasma (AIP)—both considered surrogate markers for atherosclerosis—and major adverse cardiovascular events (MACEs) in patients diagnosed with chronic coronary syndrome (CCS). We conducted a retrospective analysis, encompassing 715 consecutive patients with intermediate CCS risk, who presented at the outpatient clinic between June 2020 and August 2022. MACEs included non-fatal myocardial infarction, hospitalization for heart failure, cerebrovascular events, non-cardiac mortality, and cardiac mortality. The primary outcome was the composite occurrence of MACEs during the follow-up period. For time-to-event analysis of the primary outcome, we employed Kaplan–Meier plots and Cox proportional hazard models. The median age of the overall study population was 55 years, with a median follow-up duration of 17 months. Multivariate Cox regression analysis identified age, hypertension, Coronary Artery Disease–Reporting and Data System score, and TyG index as independent predictors of the primary outcome. Notably, individuals with high TyG levels exhibited a significantly higher primary outcome rate compared to those with low TyG levels (18.7% vs. 3.8%, *p* < 0.001). Similarly, patients with elevated TyG values demonstrated statistically higher rates of cerebrovascular events, hospitalizations for heart failure, non-fatal myocardial infarctions, non-cardiac mortality, and cardiac mortality. These findings suggest that TyG may serve as a predictive marker for adverse cardiovascular outcomes in patients with CCS.

## 1. Introduction

Coronary artery disease (CAD) is a clinical condition arising from the development of atherosclerotic plaques within the epicardial coronary arteries, regardless of whether they are obstructive or not [1,2]. Acute coronary syndromes, accounting for a substantial proportion of cardiovascular mortality, are characterized by the sudden occurrence of total or near-total thrombogenic occlusions in previously compromised coronary artery architecture [3]. Chronic coronary syndrome (CCS) represents a progressive form of CAD amenable to treatment through lifestyle modifications, medications, and invasive procedures [1,3]. The early identification of CCS patients is of paramount importance in advancing disease prevention, management, and mitigating adverse outcomes [3]. Inflammation, aberrant glucose metabolism, and lipid dysregulation all play pivotal roles in atherogenesis and thus constitute significant risk factors in CAD development [2]. Moreover, CCS patients exhibiting both abnormal glucose metabolism and dyslipidemia face an elevated risk of experiencing unfavorable cardiovascular outcomes [4,5]. The triglyceride–glucose index (TyG), a composite marker of fasting plasma glucose and triglyceride (TG) levels, is recognized as an indicator of metabolic derangements [6,7]. Another recently defined marker portraying the balance between atherogenic and anti-atherogenic factors is the atherogenic index of plasma (AIP) [8]. Numerous studies have attested to elevated TyG and AIP levels as independent risk factors for atherosclerosis and their association with an increased risk of cardiovascular events [9,10,11,12,13]. The primary objective of this study is to assess the relationship between TyG and AIP and major adverse cardiovascular events (MACEs) in intermediate-risk CCS patients.

## 2. Materials and Methods

### 2.1. Study Population

**Figure 1:**
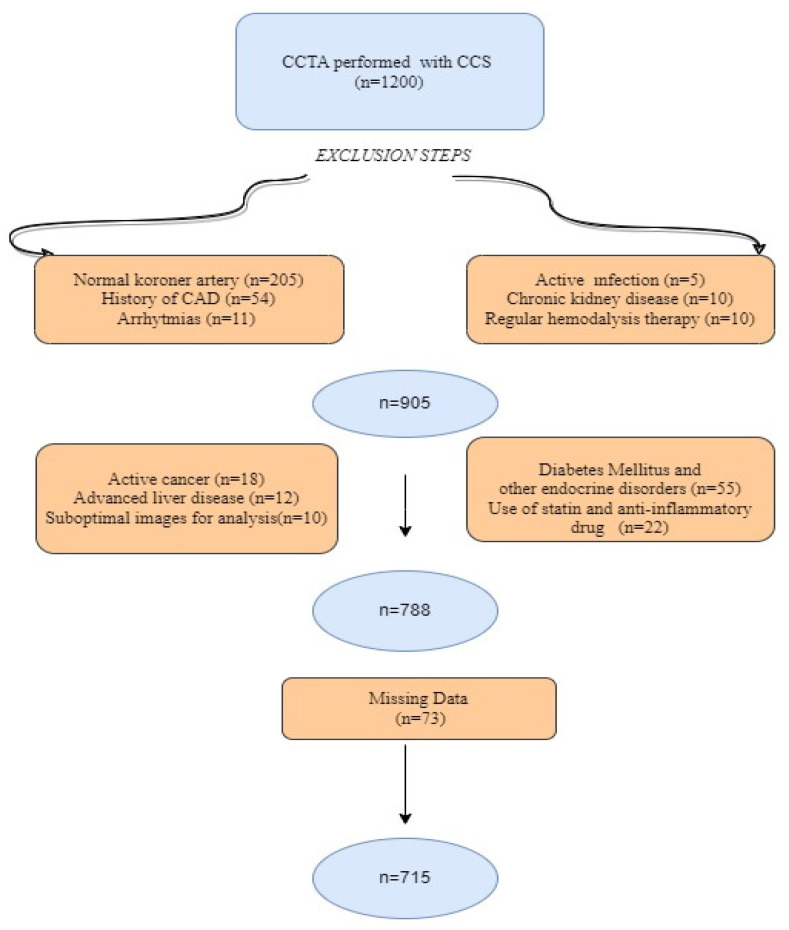
Flow chart of exclusion steps.

This retrospective observational study enrolled 1200 consecutive patients who presented at our outpatient clinic with stable angina pectoris and/or angina-equivalent symptoms and subsequently underwent coronary computed tomography angiography (CCTA) between June 2020 and August 2022. The pre-test probability scores of each patient, calculated based on factors, including age, gender, diabetes mellitus (DM), hypertension, smoking, dyslipidemia, and family history, were assessed [3]. The study included individuals with intermediate risk (pre-test probability: 15–85%) of CAD who underwent CCTA as a diagnostic procedure. In addition, the following criteria were applied to include subjects in the study: (i) those with suspected CAD due to symptoms such as stable angina or angina-equivalent symptoms like dyspnea and (ii) those with no history of CAD [3]. A total of 485 individuals were excluded from further analysis as they met at least one of the following criteria: known normal coronary artery anatomy, a history of CAD, atrial fibrillation, atrial flutter, atrioventricular block, presence of implantable intracardiac devices, severe valvular disorders, diabetes mellitus (DM) or other endocrine disorders, use of antihyperlipidemic medication within the past six months, active infection, active malignancy, advanced liver disease, a glomerular filtration rate lower than 30 mL/min/1.73 m^2^, and/or end-stage renal disease (estimated glomerular filtration rate, eGFR, less than 15 mL/min/1.73 m^2^ or receiving regular hemodialysis treatment). Initially, we performed an assessment of image quality and interpretability. Images that received a quality rating below <3 on the Likert scale were excluded from the study as they did not meet the criteria for suitable evaluation (Figure 1). Demographic, laboratory, and clinical data of the remaining 715 individuals for the final analysis were extracted from our institution’s electronic medical record system. The study was conducted in accordance with the ethical principles stated in the Declaration of Helsinki. The study protocol received approval from the institution’s ethics committee (date: 23 November 2022, decision number: 374). The requirement for written informed consent was waived due to the retrospective nature of the study.

### 2.2. Definitions and Risk Factors

At the initiation of this study, we collected baseline data involving various laboratory parameters for each participant prior to the commencement of the procedure. These parameters included a complete blood count analysis (performed using the Beckman Coulter LH 750, Fullerton, CA, USA), lipid profile, albumin, glucose levels, and other pertinent biochemical variables (measured utilizing the Cobas C7001 by Roche Diagnostics, Rotkreuz, Switzerland). To evaluate renal function, we employed the eGFR calculation method, as validated in the Modification of Diet in Renal Disease Study [14]. The presence of HT was ascertained based on participants either taking antihypertensive medication or having systolic blood pressure exceeding 140 mm Hg and/or diastolic blood pressure exceeding 90 mmHg [15]. DM was defined as a fasting glucose level ≥ 126 mg/dL, postprandial blood glucose ≥ 200 mg/dL, or the use of antidiabetic therapy [16]. Dyslipidemia was identified if at least one of the following lipid profile patterns was observed: total cholesterol level ≥ 240 mg/dL, low-density lipoprotein (LDL) level ≥ 130 mg/dL, high-density lipoprotein (HDL) level < 40 mg/dL for men or <50 mg/dL for women, and triglyceride level ≥ 150 mg/dL [16]. Body mass index (BMI) was computed as the ratio of weight in kilograms divided by the square of height in meters (kg/m^2^). Participants were classified as current smokers if they had a history of consistent smoking or had been smokers in the month preceding the study. AIP was calculated as the logarithmically transformed ratio of TG concentration to HDL-C (AIP = Log10(TG/HDL-C)) [10]. TyG was computed using the formula ln(fasting TG (mg/dL) × fasting glucose/2) [7]. In our study, these measurements were initially obtained as part of the participants’ routine clinical assessments. Subsequently, AIP and TyG values were calculated using the aforementioned formulas.

### 2.3. CCTA Scan and Data Analysis

Coronary computed tomography angiography imaging was performed at a median interval of 7 days subsequent to the clinical and laboratory assessments of the patients. All imaging data were acquired using a 640-slice CT scanner (Aquilion; Canon, Otawara-shi, Japan). To ensure objectivity and minimize potential bias, an experienced radiologist, who remained blinded to both the patients’ baseline characteristics and the study design, interpreted these images. Coronary artery plaque (CAP) is characterized as an anomalous structural formation resulting from the accumulation of lipid deposits, cholesterol, calcium, and various substances on the endothelial lining of the coronary artery, discernible in two distinct image planes [17]. The classification of CAP involved the modeling of all coronary arteries, including the left main coronary arteries, according to the American Heart Association classification. These arteries were further subdivided into 16 distinct segments, which included proximal, middle, and distal portions of the left anterior descending arteries; proximal, middle, and distal segments of the circumflex arteries; proximal, middle, and distal segments of the broad marginal branches; and proximal, middle, and distal portions of the right coronary arteries. This classification was achieved through the utilization of original axial views, thin sections, maximum intensity projections, and cross-sectional reconstructions orthogonal to the long axis of each coronary segment, each with a thickness of 0.5 mm [18]. All identified plaques were meticulously documented based on their corresponding segments. The most severe stenosis observed in any coronary vessel was assessed and graded in accordance with the Coronary Artery Disease–Reporting and Data System (CAD-RADS) score. The grading scale contained CAD-RADS 0 (denoting 0% stenosis and an absence of plaque), 1 (indicating 1 to 24% stenosis or a non-stenotic plaque with positive remodeling), 2 (reflecting 25 to 49% stenosis, mild stenosis), 3 (signifying 50 to 69% stenosis, moderate stenosis), 4a (representing 70 to 99% stenosis in 1 or 2 vessels), 4b (indicating 70 to 99% stenosis in 3 vessels or left main stenosis exceeding 50%), and 5 (denoting total occlusion) [19].

### 2.4. Medical Treatment and Follow-Up

Patients diagnosed with CAD following comprehensive clinical, laboratory, and CCTA assessments received treatment in accordance with the 2019 European Society of Cardiology guidelines for the diagnosis and management of CCS recommendations [3]. In instances where CAD was concurrent with additional risk factors, such as newly diagnosed prediabetes and hyperlipidemia, our medical team collaborated closely with relevant specialists and departments to initiate treatment based on consensus and best practices. Patient follow-up and clinical event analysis were meticulously conducted utilizing both the national health system and our hospital’s automated system. In cases where complete information was not readily accessible, we adopted an approach involving direct communication with patients via telephone to obtain essential details regarding their clinical status.

### 2.5. Outcomes

The primary outcome was defined as the composite of parameters comprising MACEs that occurred during the follow-up period after discharge. MACEs included the following components: non-fatal myocardial infarction (MI), hospitalization for heart failure, cerebrovascular events, non-cardiac mortality, and cardiac mortality. Secondary outcomes were defined as each individual component of MACEs. Cardiac mortality was specifically characterized as death primarily attributed to acute MI, congestive heart failure, malignant arrhythmia, or other structural heart diseases.

### 2.6. Statistical Analysis

We assessed the normality of continuous variables through a combination of analytical methods, specifically employing the Kolmogorov–Smirnov test, and visual methods such as the examination of histograms and probability plots. Continuous covariates were presented as either mean ± standard deviation or median (interquartile range: IQR 25–75), and their distribution was evaluated using either the Mann–Whitney U test or the independent Student’s *t*-test, contingent on their adherence to normality assumptions. Categorical variables underwent analysis using Pearson’s chi-square test or Fisher’s exact test, and the results were reported in terms of counts (n) and percentages (%). In the context of time-to-event analysis for our primary outcome, we utilized Kaplan–Meier plots, the log-rank test, and Cox proportional hazard models. Initially, we constructed a foundational Cox proportional hazard regression model exclusively incorporating adjustment variables. The selection of these variables for the base model was guided by univariate Cox regression analysis and included age, hypertension, hyperlipidemia, C-reactive protein (CRP), and the CAD-RADS (3, 4a, 4b, 5) score. Subsequently, to create comprehensive models, we separately introduced TyG and AIP into the base model, thereby generating two distinct full models. Model performance was assessed using various metrics, including the Likelihood ratio X^2^ (higher values indicating a better fit), Akaike information criteria (lower values signifying a superior fit), R^2^ (higher values indicating an improved fit), and C-index (higher values reflecting enhanced discrimination). Furthermore, to establish cutpoints in time-to-event data, we employed maximally selected rank statistics. This allowed us to categorize observations into two groups based on TyG and AIP for mortality prediction. We compared the TyG cutpoints using the log-rank test and scrutinized the discriminatory and additive value of TyG and AIP. Throughout our study, statistical significance was defined as a two-tailed *p*-value below 0.05. All statistical analyses were conducted using R version 4.22 software (Vienna, Austria) in conjunction with the “DescTools,” “survminer,” “ggplot2,” and “maxstat” packages.

## 3. Results

### 3.1. Baseline Characteristics

The total study cohort comprised 715 participants, with a median age of 55 years (IQR: 49–62), of which 415 (58%) were male. The median follow-up duration lasted 17 months (IQR: 15–22). During this period, the overall incidence of MACEs was 9.2% (n = 66). The median TyG value for the entire study population was calculated as TyG 9.21 (IQR: 8.98–9.56), while the AIP was found to be AIP 0.25 (IQR: 0.12–0.38). To categorize patients based on their TyG levels, a maximal rank statistics approach was employed, resulting in two distinct groups: the “low TyG” group with levels ≤ 9.59 and the “high TyG” group with levels > 9.59. Subsequently, a comprehensive comparative analysis of their baseline characteristics was conducted. No significant difference was noted in terms of the timing of clinical evaluations and laboratory tests relative to CCTA acquisition between the high- and low-TyG-index groups (7 days (IQR: 5–14) vs. 9 days (IQR: 7–16), *p* = 0.567, respectively). However, the high-TyG group exhibited a higher median age (57 (IQR: 50–65) vs. 54 (IQR: 48–61), *p* < 0.001, respectively), a greater prevalence of hypertension (*p* = 0.012), and a higher frequency of CAD-RADS 3, 4a, 4b, and 5 scores (*p* < 0.001). Male gender, smoking status, family history of CAD, medical treatment, and BMI, on the other hand, were comparable between the two groups. The high-TyG group also demonstrated elevated levels of CRP, creatinine, albumin, LDL, TG, white blood cells, platelets, and monocytes (*p* < 0.05 for all). A detailed breakdown of the demographic, clinical, and laboratory characteristics of the study population according to TyG levels is presented in Table 1.

### 3.2. Primary and Secondary Outcomes

**Figure 2:**
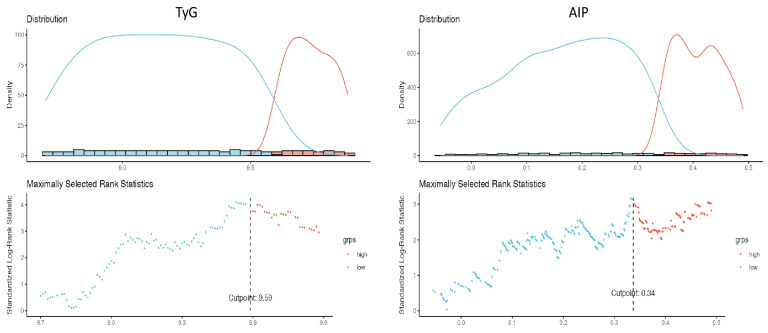
Cutpoint plot with the maximally selected rank test.

Throughout the follow-up period, the primary outcome, characterized by the occurrence of MACEs, was more frequently observed in the high-TyG group compared to the low-TyG group (18.7% vs. 3.8%, *p* < 0.001, respectively) (Figure 2). Additionally, patients with elevated TyG levels exhibited higher incidence rates of cerebrovascular events (2.7% vs. 0.7%, *p* < 0.001), hospitalization for heart failure (3.4% vs. 0.9%, *p* = 0.003), non-fatal myocardial infarction (MI) (5.3% vs. 0.7%, *p* < 0.001), non-cardiac mortality (3.4% vs. 0.4%, *p* < 0.001), and cardiac mortality (3.8% vs. 1.1%, *p* = 0.002) (Table 2).

### 3.3. Independent Predictors of MACEs at Follow-Up

The Cox proportional hazards model was employed to investigate the relationship between MACEs and TyG and AIP. In the univariate analysis, significant associations were observed between MACEs and TyG (hazard ratio (HR): 4.29, 95% confidence interval (CI) 3.11–6.79, *p* < 0.001) as well as AIP (HR: 3.42, 95% CI 3.00–5.68, *p* < 0.001) (Table 3). Even after adjusting for several risk factors and important clinical variables in the multivariate model, TyG remained an independent predictor of MACEs (HR: 2.11, 95% CI 1.55–3.12, *p* = 0.003), while AIP did not reach statistical significance (*p* = 0.091). Furthermore, age, hypertension, and CAD-RADS 3, 4a, 4b, and 5 scores retained their status as independent predictors in both adjusted models (Table 4).

### 3.4. Diagnostic Performance of TyG and AIP for Cardiovascular Outcomes

**Figure 3:**
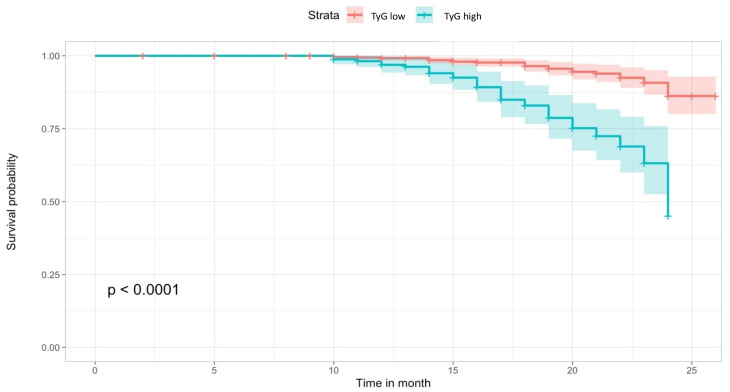
Kaplan–Meier curve for survival analysis of MACE-free survival.

We employed maximally selected rank statistics to determine optimal cut-off points in the time-to-event data, thereby categorizing observations into two groups based on TyG and AIP for mortality estimation (TyG: low ≤ 9.59 vs. TyG high > 9.59, AIP: low ≤ 0.34 vs. high >0.34) (Figure 2). The cumulative hazard on the survival curve indicated a higher mortality rate in the high-TyG group (log-rank test, *p*-value < 0.001) (Figure 3). The model that exhibited the most robust predictive performance (as assessed by Likelihood ratio X2 and R2) and discrimination incorporated TyG as a continuous variable. The Harrel’s C-index of the full models, which included TyG continuous scores, was significantly higher than the AIP model (Table 5).

## 4. Discussion

The results of this study imply that the TyG and AIP indexes hold the potential to predict outcomes and assist in the prognostic stratification of patients with CCS. Significantly, TyG emerged as a more effective predictor of MACEs when compared to AIP. Patients with elevated TyG levels demonstrated a nearly twofold increase in the risk of experiencing MACEs during the follow-up period. Furthermore, factors such as age, hypertension, and the severity of CAD were also identified as independent predictors of MACE.

The AIP is a recently developed prognostic index that is closely associated with plasma TG and HDL-C levels [20]. Elevated AIP levels indicate high TG and/or low HDL-C levels, which in turn signify a shift towards smaller, denser LDL particles and an increase in the concentration of small, dense LDL (sd-LDL) particles [8,19,20]. AIP has been demonstrated to accurately reflect levels of sd-LDL, which are highly atherogenic due to their increased susceptibility to oxidation and their association with oxidized apoprotein B and LDL-C. Furthermore, sd-LDL particles activate oxygen radicals, promote lipid peroxidation, and induce the expression of adhesion molecules in endothelial cells, all of which contribute to endothelial dysfunction [21,22]. In contrast, HDL-C plays a crucial role in transporting peripheral cholesterol to the liver and possesses antioxidant properties [1]. These insights underscore the accumulating evidence suggesting that AIP may serve as a significant determinant of atherosclerosis and cardiovascular diseases (CVD) [8,9,10,20,21,22].

Prior research conducted in various populations, including postmenopausal women [23], diabetic patients with kidney disease [24], and individuals under investigation for subclinical atherosclerosis [25], consistently identified AIP as an independent prognostic index. In our study, we aimed to assess its robust independent predictive power in conjunction with major risk factors in patients diagnosed with stable CAD. While we observed a higher rate of MACEs at higher AIP cut-off values during rank statistical analysis, this difference did not achieve statistical significance in multivariate analysis. Several factors could account for this outcome. Firstly, our patient cohort was relatively stable, and the follow-up duration was relatively shorter, which may have contributed to the lower MACE rate and the absence of statistical significance. Secondly, previous studies may have focused on patient populations with a greater burden of atherosclerosis, where the predictive capacity of the AIP index is enhanced, especially in groups with impaired cholesterol metabolism such as dyslipidemia, diabetes, and metabolic disorders [8,9,10].

The TyG index, which combines measurements of TG and fasting plasma glucose, has gained significant recognition as a valuable tool for assessing insulin resistance [6]. Recent studies have shown that TyG may outperform the homeostatic model assessment for insulin resistance (HOMA-IR) in this context and can serve as a predictor for CAD and adverse cardiovascular events [12,26,27]. TyG’s predictive value for CVD likely stems from its ability to reflect disorders in both glucose and lipid metabolism [26]. Individuals with insulin resistance are at an elevated risk of CVD due to heightened proinflammatory responses and ventricular and vascular dysfunction associated with dense atherosclerotic plaques [28,29]. Furthermore, alterations in systemic lipid metabolism resulting from insulin resistance can lead to vascular endothelial damage and increased inflammation, potentially accelerating the rupture of vulnerable plaques [29]. Notably, the Prospective Urban Rural Epidemiology (PURE) study linked high carbohydrate intake, which can elevate plasma TG and glucose levels, to an increased overall risk of mortality [30]. Consequently, TyG may offer valuable insights into the shared role of glucose and lipid metabolism in predicting CVD risk.

Numerous studies have also established a robust association between TyG and the severity of CAD [12,13,31,32,33,34,35]. In our study, TyG emerged as a robust predictor of mid-term adverse outcomes in patients with CCS. Additionally, our research demonstrated that TyG exhibited superior discriminative capacity compared to the AIP. The diligent use of TyG and AIP for identifying CCS patients at risk for unfavorable outcomes holds the potential to enhance medical care and reduce the incidence of acute adverse cardiac events. During the follow-up period, some patients initiated oral antidiabetic and antihyperlipidemic treatments due to newly diagnosed metabolic disorders and prediabetes. While our study did not reveal statistically significant impacts of these medications on MACEs, several considerations are important. The relatively small number of patients with metabolic dysregulation, who were evenly distributed between the high- and low-TyG groups, and the relatively short duration of follow-up may have limited the detection of the potential efficacy of these drugs. Importantly, prior research has emphasized the substantial benefits of antidiabetic and antihyperlipidemic medications in improving the prognosis of individuals with chronic CAD [3,4,5,6,7,8,9,10,11,12,13,14,15,16,17,18,19,20,21,22,23,24,25,26,27,28,29,30,31,32,33,34,35,36]. We also hypothesize that these medications might exert a more pronounced effect in more focused and comparative studies, particularly when compared with a control group.

Our study has revealed a significant association between an elevated TyG index and the risk of MACEs. Importantly, this increased risk of MACEs remained significant even when accounting for traditional cardiovascular risk factors, comorbidity burden, disease severity, and medications. Consequently, our findings align with previous research that has established a strong link between the TyG index and cardiovascular events in patients with CAD [13,23,31]. Moreover, in our study, we established the optimal threshold for the TyG index to predict MACEs, which we found to be 9.59 using rank statistics. Additionally, we integrated the TyG index with established risk factors for MACEs and computed a C-statistic, highlighting the significant discriminatory power of our model. These findings emphasize the potential of the TyG index to improve the stratification of cardiovascular risk. The inclusion of the TyG index into routine clinical diagnostic models holds promise for improving the precision of identifying patients at heightened cardiovascular risk, thereby enabling more targeted treatment and prevention strategies.

To the best of our knowledge, our study represents the largest cohort investigation to date assessing MACEs in patients diagnosed with CCS using the TyG index in conjunction with CCTA. An important aspect of our study is the precise timing of clinical evaluations and laboratory tests concerning the acquisition of CCTA data. We based our results on assessments conducted shortly before the CCTA acquisition to ensure the collection of the most accurate and up-to-date data regarding the cardiovascular status of our participants. The prognostic value of CCTA has received extensive support from various ethnic groups and numerous previous studies. Its selective use in primary prevention settings is endorsed by clinical guidelines [3,37]. CCTA provides a more comprehensive evaluation that goes beyond merely detecting the presence or severity of stenosis. It offers superior insights into plaque structure, vulnerability, and high-risk plaque classification compared to conventional angiography [36]. With advancing technology, CCTA has started to play a crucial role in guiding both invasive and noninvasive treatments for coronary artery disease by providing physiological assessments. However, concerns persist regarding its widespread use, primarily due to potential radiation side effects and the risk of contrast-induced nephropathy [36,37]. As our study has also highlighted, an attractive, cost-effective, and reliable approach may involve combining the TyG and AIP indices with CCTA findings. This combination could serve as a significant parameter for predicting atherosclerosis and MACEs, potentially offering a valuable tool for risk assessment and management in clinical practice.

## 5. Study Limitation

This study, while offering valuable insights, is not without its limitations. Firstly, it is important to acknowledge that this research is retrospective and non-randomized in design. This inherent limitation may affect the ability to establish causal relationships and requires a cautious interpretation of the findings. Secondly, the sample size, while representative, may not be sufficiently large to capture the full spectrum of variability within the population. Additionally, the relatively short follow-up duration may have limited the observation of long-term outcomes and trends. Thirdly, the relatively low number of participants who experienced adverse events during the study period raises considerations regarding the generalizability of our findings. Future studies with larger cohorts and longer follow-up durations are needed to further validate and refine these associations. Fourthly, it is important to note that our models did not account for additional potentially confounding factors, such as dietary preferences, lifestyle choices, or exercise routines. These factors can play a significant role in cardiovascular health and may have influenced our results. In summary, while our study contributes important insights, these limitations should be taken into consideration when interpreting the results, and further research is warranted to build upon and confirm these findings.

## 6. Conclusions

While prior research has established a link between TyG and AIP and cardiovascular risk, our study provides further evidence that TyG and AIP may indeed be related to MACEs in patients with CCS. Importantly, our findings suggest that TyG may offer superior discriminative performance compared to AIP in predicting MACEs during the follow-up period. These noninvasive and easily calculated clinical markers hold promise in estimating unfavorable outcomes and may contribute to improved prognostic assessments in clinical practice.

## Figures and Tables

**Table 1:** Baseline characteristics of the study population.

Variables	TyG			*p*-Value *
	Overall	Low	High	
		(≤9.59)	(>9.59)	
	(n = 715)	(n = 453)	(n = 262)	
Demographic features and risk factors				
Age; median, (IQR)	55 (49–62)	54 (48–61)	57 (50–65)	<0.001
Male; n (%)	415 (58)	252 (55.2)	163 (62.2)	0.069
Hypertension; n (%)	305 (42.6)	176 (38.8)	129 (49.2)	0.012
HL; n (%)	165 (24.5)	101 (23.7)	64 (25.9)	0.511
Smoking; n (%)	41 (5.7)	23 (5.2)	18 (6.8)	0.255
Family history of CAD; n (%)	109 (15.2)	73 (16.1)	36 (13.7)	0.364
BMI	23.6 (22.7–24.6)	23.7 (22.5–24.4)	23.7 (23.0–24.3)	0.315
Angiographic results, n (%)				
CAD-RADS (3, 4a, 4b, 5); n (%)	257 (35.9)	58 (12.8)	199 (75.9)	<0.001
Laboratory findings				
Total cholesterol, mmol/L; Median (IQR)	4.49 (3.91–5.25)	4.23 (3.72–4.70)	5.13 (4.60–5.84)	0.567
Triglyceride, mmol/L; Median (IQR)	2.09 (1.62–2.63)	1.74(1.21–2.10)	2.77 (2.41–3.44)	<0.001
HDL-C, mmol/L; Median (IQR)	1.14 (1.02–1.31)	1.19(1.03–1.31)	1.12(0.98–1.31)	0.001
LDL-C, mmol/L; Median (IQR)	3.03 (2.31–3.60)	2.5 (2.0–3.4)	3.2 (2.4–3.7)	<0.001
Creatinine, mg/dL; Median (IQR)	0.81 (0.72–0.93)	0.7 (0.6–0.9)	0.83 (0.74–0.91)	0.014
e-GFR, mL/min/1.73 m^2^; Median (IQR)	92 (85–101)	93 (87–102)	91 (82–101)	0.003
Glucose, mg/dL; Median (IQR)	114 (102–129)	108 (96–122)	125 (114–157)	<0.001
WBC, 10^3^/dL; Median (IQR)	7.7 (6.6–9.3)	7.4 (6.5–8.9)	8.6 (7.0–9.9)	<0.001
Hemoglobin, g/dL; Median (IQR)	13.7 (12.7–14.8)	13.9 (12.6–14.9)	14 (12.9–15.3)	0.341
Platelet count, 10^3^/dL; Median (IQR)	260 (231–294)	253 (225–284)	275 (251–305)	<0.001
Lymphocyte, cells/µL, Median (IQR)	2.2 (1.9–2.5)	2.3 (1.8–2.5)	2.1 (1.9–2.3)	<0.001
Monocytes, cells/µL; Median (IQR)	0.61 (0.53–0.75)	0.6 (0.51–0.72)	0.65 (0.53–0.81)	0.003
Neutrophils, cells/µL; Median (IQR)	4.4 (3.8–5.4)	4.2 (3.7–4.8)	5.2 (4.5–6.5)	<0.001
CRP, mg/L; Median (IQR)	5.4 (4.2–7.9)	4.9 (3.9–6.1)	7.6 (5.5–9.3)	<0.001
Albumin, g/dl; Median (IQR)	4.6 (4.4–4.7)	4.4 (4.4–4.7)	4.6 (4.5–4.7)	0.003
Medications prescribed at discharge, n (%)				
Antiplatelets, n (%)	545 (76.2)	340 (75.0)	205 (78.2)	0.901
B-blockers, n (%)	350 (48.9)	218 (48.1)	132 (50.3)	0.801
ACEIs or ARBs	210 (29.3)	125 (27.5)	85 (32.4)	0.855
OAD, n (%)	183 (25.5)	119 (26.2)	64 (24.4)	0.948
Antihyperlipidemic, n (%)	165 (23.0)	104 (22.9)	61 (23.2)	0.645
Statins, n (%)	155 (21.6)	98 (21.6)	57 (21.7)	0.763
Fenofibrate, n (%)	10 (1.3)	6 (1.3)	4 (1.5)	0.851

Values are presented as numbers (n) and percentages (%), mean ± standard deviation, or median (interquartile range, 25th–75th percentiles). For continuous data, the *p*-value was calculated using the independent samples *t*-test or the Mann–Whitney U-test, and for categorical variables, the chi-square test or Fisher’s exact test, as appropriate. * *p* < 0.05 was considered statistically significant. Abbreviations: ACEIs, angiotensin-converting enzyme inhibitors; ARBs, angiotensin receptor blockers; CAD, coronary artery disease; CAD-RADS, Coronary Artery Disease–Reporting and Data System; CRP, C-reactive protein; e-GFR, estimated glomerular filtration rate; HDL-C, high-density lipoprotein cholesterol; HL, hyperlipidemia; IQR, interquartile range; LDL-C, low-density lipoprotein cholesterol; OAD, oral antidiabetic drug; WBC, white blood cell.

**Table 2:** Comparison of primary and secondary outcomes between low- and high-TyG groups.

	TyG			
	Overall	Low Group	High Group	*p*-Value *
		(≤9.59)	(>9.59)	
The primary outcome, n (%)				
MACE	66 (9.2)	17 (3.8)	49 (18.7)	<0.001
Secondary outcomes, n (%)				
Cerebrovascular events	10 (1.4)	3 (0.7)	7 (2.7)	<0.001
Hospitalization for heart failure	13 (1.8)	4 (0.9)	9 (3.4)	0.003
Non-fatal MI	17 (2.4)	3 (0.7)	14 (5.3)	<0.001
Non-cardiac Mortality	11 (1.5)	2 (0.4)	9 (3.4)	<0.001
Cardiac Mortality	15 (2.1)	5 (1.1)	10 (3.8)	0.002

Data are given as numbers (n) and percentages (%). The chi-square test or Fisher’s exact test was performed. * *p* < 0.05 was considered statistically significant. Abbreviations: MACEs, major adverse cardiovascular events.

**Table 3:** Univariate Cox regression analysis for MACE at follow-up.

	Unadjusted		
Variable	HR	95% CI	*p*-Value *
Age	1.03	1.02–1.07	<0.001
Gender (male reference)	0.63	0.44–1.01	0.056
Hypertension	2.29	1.47–3.35	<0.001
Hyperlipidemia	1.46	1.12–2.35	0.021
Hemoglobin	0.89	0.81–0.97	0.119
Creatinine, mg/dL	0.96	0.41–3.75	0.934
CRP; mg/dL	1.03	1.01–1.12	<0.001
Family history of CAD	1.10	0.67–1.87	0.599
Smoking	1.15	0.81–1.57	0.311
LDL-C, mmol/L	0.89	0.71–1.09	0.337
HDL-C, mmol/L	0.66	0.53–0.72	0.289
OAD, n (%)	0.74	0.61–0.91	0.323
Antihyperlipidemic, n (%)	0.91	0.72–0.99	0.453
BMI	1.01	0.90–1.27	0.396
CAD-RADS (3, 4a, 4b, 5)	4.74	3.35–7.45	<0.001
TyG index	4.29	3.11–6.79	<0.001
AIP	3.42	3.00–5.68	<0.001

* *p* < 0.05 was considered statistically significant. Abbreviations: AIP, atherogenic index of plasma; BMI, Body mass index, CAD, coronary artery disease; CAD-RADS, Coronary Artery Disease–Reporting and Data System; CRP, C-reactive protein; HDL-C, high-density lipoprotein cholesterol; HR, hazard ratio; LDL-C, low-density lipoprotein cholesterol; OAD, oral antidiabetic drug; TyG, triglyceride–glucose index.

**Table 4:** Multivariate Cox proportional hazard regression models to predict MACE.

	Adjusted Model 1			Adjusted Model 2		
Variable	HR	95% CI	*p*-Value	HR	95% CI	*p*-Value *
Age	1.05	1.01–1.09	<0.001	1.04	1.02–1.06	<0.001
Hypertension	1.03	1.02–3.58	0.041	1.46	1.00–2.41	0.045
Hyperlipidemia	1.07	0.79–2.41	0.286	1.32	0.78–1.97	0.374
CRP; mg/dL	1.06	0.93–1.09	0.121	1.09	0.87–1.21	0.261
CAD-RADS (3, 4a, 4b, 5)	2.87	1.75–5.11	<0.001	2.73	1.57–4.85	0.002
AIP	2.79	0.873–7.8	0.091	-	-	-
TyG index	-	-	-	2.11	1.55–3.12	0.003

* *p* < 0.05 was considered statistically significant. Abbreviations: AIP, atherogenic index of plasma; CRP, C-reactive protein; TyG, triglyceride–glucose index; HR, hazard ratio.

**Table 5:** Model performance comparison between TyG and AIP full model.

	Likelihood Ratio X^2^	Nagelkerke’s Adjusted R^2^	Akaike Information Criteria	Harrel’s C-Index (Model AUC)
TyG	23.07	0.412	422	0.688
AIP	14.32	0.332	435	0.660

## Data Availability

Data are available on request due to privacy and ethical restrictions.
